# Modeling Exposure–Mortality Relationships for Indoor Heat

**DOI:** 10.1111/risa.70395

**Published:** 2026-10-21

**Authors:** Andrew Ibbetson, Charles Simpson, Giorgos Petrou, Eleni Oikonomou, Anna Mavrogianni, Rajat Gupta, Mike Davies, Ai Milojevic

**Affiliations:** ^1^ Institute for Environmental Design and Engineering, The Bartlett School of Environment, Energy and Resources University College London London UK; ^2^ Low Carbon Building Research Group School of Architecture, Oxford Brookes University Oxford UK; ^3^ Department of Public Health, Environments and Society, Faculty of Public Health and Policy London School of Hygiene & Tropical Medicine London UK

**Keywords:** heat health, heat‐related mortality, indoor heat, mathematical modeling, overheating

## Abstract

Most individuals spend the majority of their time indoors. However, epidemiological evidence of the association between heat and mortality is dominated by studies using outdoor temperature as a proxy for personal exposure. To demonstrate how an exposure–response relationship between indoor heat and mortality may relate to that between outdoor heat and mortality, we develop a novel mathematical modeling approach. This approachuses the relationship between regional daily average indoor and outdoor temperature to map the exposure–response relationship for outdoor heat, estimated by epidemiological studies, to indoor heat. Due to the modifying effect of buildings on heat exposure, we show that a change in indoor temperature may be associated with a greater change in mortality than an equal change in outdoor temperature. This has implications for researchers seeking to assess the health impact of changes in indoor heat exposure. Furthermore, it provides a new theoretical  framework for interpreting potential differences in the temperature‐mortality relationship of indoor and outdoor heat exposure in epidemiological studies.

## 1 Introduction

Epidemiological evidence of the association between heat and mortality is dominated by studies using outdoor temperature as a proxy for personal exposure (Gasparrini et al. 2022). However, studies demonstrate that individuals in high‐income countries with temperate climates spend 90% of their time indoors (Klepeis et al. 2001; Schweizer et al. 2007), implying that the mortality effects of outdoor temperature are strongly mediated by the indoor environment. This is particularly the case for those most at risk of heat‐related mortality, such as the elderly and those living in care homes, who, due to their frailty and lack of mobility, spend nearly all their time indoors (Almeida‐Silva et al. 2014).

Extensive research has demonstrated the ability of building interventions, such as ventilation and shading, to reduce indoor overheating risks in a variety of settings (Coley and Kershaw 2010; Gupta and Gregg 2012; Mavrogianni et al. 2012; Oikonomou et al. 2025; Porritt et al. 2012). This has led to several studies that estimate the potential reduction in heat‐related mortality due to such interventions (Ibbetson et al. 2025; Taylor et al. 2021). In the absence of an exposure–response relationship between indoor temperature and mortality, these studies have relied on the assumption that changes in indoor temperature will have an effect on mortality equivalent to moving an individual up or down the exposure–response relationship for *outdoor* temperature by a corresponding amount. The validity of this assumption remains unclear.

Analyses of the relationship between daily outdoor and indoor temperature of a diverse range of buildings, in a variety of climates, indicate that an increase in outdoor temperature in the warm season is typically associated with a smaller increase in indoor temperature (Drury 2024; Nguyen et al. 2014; Nguyen and Dockery 2016; Nicol et al. 2020; Rupp et al. 2021; Tamerius et al. 2013). For example, in a representative sample of 616living rooms in England during the summer of 2018, the linear slope between 2‐day mean maximum outdoor temperature for the previous 2 days and daily mean indoor temperature was 0.40 [SD = 0.12] (DESNZ and Li 2024). These findings may suggest that, if indoor temperature strongly mediates the association between outdoor temperature and mortality, a change in indoor temperature is associated with a greater change in mortality than an equivalent change in outdoor temperature. A similar observation has been made for indoor air pollution from outdoor sources, using an argument based on the infiltration rate of buildings, which is typically less than unity (Ji and Zhao 2015; Milner et al. 2017). Thus, as the concentration of outdoor air pollution rises, the concentration of indoor air pollution of outdoor origin increases by a lesser amount due to the limited air permeability of the building.

To our knowledge, only three epidemiological studies have estimated an exposure–response relationship between indoor temperature and mortality (Huang et al. 2025; O'Lenick et al. 2020; Ragettli et al. 2024). All these studies used building physics modeling or predictive models based on monitored data to estimate daily indoor temperature exposure at an individual or population level. The estimated exposures to indoor temperature were then linked with daily mortality data, and the relationship between them was analyzed using regression. O'Lenick et al. (2020) used an individual‐level case‐crossover design with a linear, no‐threshold model to assess the association between circulatory deaths and daily maximum temperature for the above 65 years old (*n* = 18,330) during the summer period 2000–2015 in Houston, TX. They reported an odds ratio (OR) of 1.16, 95% CI [1.03, 1.30] per 5°C increase. Ragettli et al. (2024) applied a time‐stratified case‐crossover design with a distributed lag nonlinear model (DLNM) to individuals of the Swiss population (*n* = 320,366) for the summer period 2003–2016. They estimated ORs of all‐cause mortality in the extreme heat, as the change in mortality risk from MMT to the 98th percentile of the temperature distribution during the study period, for outdoor (33°C vs. 22.7°C) and indoor temperature separately (28°C vs. 22°C). Cumulative OR over lag 0–7 days for outdoor temperature was 1.21, 95% CI [1.17, 1.25], and OR on the day for indoor temperature was 1.17 [1.15, 1.20]. For indoor temperature–mortality associations, delayed impacts were not modeled because the indoor temperature levels were simulated using the temperature levels up to 14 days before death. Huang et al. (2025), using a DLNM and regional‐level data from 364 districts in China during 2006–2017, estimated daily mean heat thresholds for all‐cause mortality of 27.5°C and 24.0°C for indoor and outdoor temperature, respectively. Above heat threshold, the risk of mortality increased by 3.0% [2.9–3.2] per 1°C increase in outdoor temperature versus 5.5% [5.0–6.0] for indoor temperature. Thus, the results of both Huang et al. (2025) and Ragettli et al. (2024) indicate that, above their respective heat thresholds, a change in indoor temperature may be associated with a greater change in mortality than an equivalent change in outdoor temperature.

More studies are needed, but their production is constrained by a lack of high spatiotemporal resolution indoor temperature data. This is because measuring indoor temperatures at scale is typically unfeasible and modeling them at high resolution is difficult due to their large variation according to a dwelling's physical characteristics, occupancy, and occupant behavior (Mavrogianni et al. 2012). As a result, studies that model indoor temperature exposures often produce temperature estimates for a restricted set of building archetypes that are chosen to represent the typical exposure of the population (Huang et al. 2025; O'Lenick et al. 2020; Ragettli et al. 2024). In doing so, they may obscure underlying heterogeneity in indoor temperature exposure. Use of these exposures to estimate the association of indoor temperature with mortality can therefore lead to exposure misclassification that reduces statistical power, decreases the ability to identify thresholds, and alters the shape of the exposure–response relationship (Rhomberg et al. 2011). Furthermore, if these archetypes are not carefully selected to ensure that they are truly representative, there may be systematic error in the modeled indoor temperatures that biases the estimated exposure–response relationship. Given these challenges, researchers and decision‐makers are likely to benefit from theoretical advancements that indicate the nature of the mortality risk posed by indoor temperatures.

In this article, we describe the development of a novel mathematical model that uses the indoor–outdoor temperature relationship to map the exposure–response relationship between outdoor heat and mortality, estimated by epidemiological studies, to indoor heat. Using this model and a mathematical proof, we aim to answer the question: Why does the exposure–response relationship between indoor heat and mortality appear to be steeper than that between outdoor heat and mortality?

## 2 Preliminaries

Our model relies on several assumptions which we describe below using the notation summarized in Table 1.

**TABLE 1:** Mathematical notation.

Symbol	Description
f	The function describing the exposure–response relationship between outdoor temperature and mortality
g	The function describing the exposure–response relationship between indoor temperature and mortality
h	The relationship between outdoor temperature, $T_{\mathrm{out}}$, and the mean threshold truncated indoor temperature, $\bar{T_{\mathrm{in}}^{+}}$
k	The relative risk of heat‐related mortality due to *indoor* temperature when $T_{\mathrm{out}}=T_{\mathrm{out}}^{*}$
${\hat{\mathrm{RR}}}_{\mathrm{in}}$	The relative risk of heat‐related mortality for a 1°C increase in regional daily mean indoor temperature, $T_{\mathrm{in}}$, above the indoor heat mortality threshold, $T_{\mathrm{in}}^{*}$
${\hat{\mathrm{RR}}}_{\mathrm{out}}$	The relative risk of heat‐related mortality for a 1°C increase in regional daily mean outdoor temperature, $T_{\mathrm{out}}$, above the outdoor heat mortality threshold, $T_{\mathrm{out}}^{*}$
$T_{\mathrm{in}}$	Regional daily mean indoor temperature
$T_{\mathrm{in}}^{*}$	Indoor heat mortality threshold
$T_{\mathrm{in}}^{+}$	Threshold truncated indoor temperature
$\bar{T_{\mathrm{in}}^{+}}$	The mean threshold truncated indoor temperature
$T_{\mathrm{out}}$	Regional daily mean outdoor temperature
$T_{\mathrm{out}}^{*}$	Outdoor heat mortality threshold

First, in line with epidemiological studies, we assume that the exposure–response relationship between outdoor temperature and mortality is described using a function, f, of the form

(1) $$f\left(T_{\mathrm{out}}\right)=\left\{\begin{array}{c} {{\hat{\mathrm{RR}}}_{\mathrm{out}}}^{\left(T_{\mathrm{out}}-T_{\mathrm{out}}^{*}\right)},\;T_{\mathrm{out}}\geq T_{\mathrm{out}}^{*}\\ 1,\;otherwise, \end{array}\right.$$

where ${\hat{\mathrm{RR}}}_{\mathrm{out}}$ is the relative risk of heat‐related mortality for a 1°C increase in regional daily mean outdoor temperature, $T_{\mathrm{out}}$, above the outdoor heat mortality threshold, $T_{\mathrm{out}}^{*}$.

Second, we assume that the exposure–response relationship between indoor temperature and mortality is described using a function, g, of the form

(2) $$g\left(T_{\mathrm{in}}\right)=\left\{\begin{array}{c} {{\hat{\mathrm{RR}}}_{\mathrm{in}}}^{\left(T_{\mathrm{in}}-T_{\mathrm{in}}^{*}\right)},\;T_{\mathrm{in}}\geq T_{\mathrm{in}}^{*}\\ 1,\;otherwise, \end{array}\right.$$

where ${\hat{\mathrm{RR}}}_{\mathrm{in}}$ is the relative risk of heat‐related mortality for a 1°C increase in regional daily mean indoor temperature, $T_{\mathrm{in}}$, above the indoor heat mortality threshold, $T_{\mathrm{in}}^{*}$.

Third, for a given regional outdoor temperature, $T_{\mathrm{out}}$, we assume that there is day‐to‐day variation in regional indoor temperature, $T_{\mathrm{in}}$. This may reflect, among other factors, differences in meteorological conditions or the behavior and occupancy patterns of residents. Therefore, for a given outdoor temperature, we denote the mean relative risk of heat‐related mortality due to indoor temperature as $\bar{g(T_{\mathrm{in}})}$.

Fourth, we assume that indoor temperature fully mediates the association between outdoor temperature and mortality, such that

(3) $$f\left(T_{\mathrm{out}}\right)=\frac{\bar{g\left(T_{\mathrm{in}}\right)}}{k},\;T_{\mathrm{out}}\geq T_{\mathrm{out}}^{*},$$

where k is the relative risk of heat‐related mortality due to *indoor* temperature when $T_{\mathrm{out}}=T_{\mathrm{out}}^{*}$. The constant k allows for the possibility that indoor temperatures may exceed the indoor heat threshold when $T_{\mathrm{out}}\leq T_{\mathrm{out}}^{*}$. For example, assuming that: (a) there is no variation in regional indoor temperature for a given outdoor temperature $\bar{g(T_{\mathrm{in}})}=g\left(T_{\mathrm{in}}\right)$ and (b) $T_{\mathrm{in}}\geq T_{\mathrm{in}}^{*}$ when $T_{\mathrm{out}}=T_{\mathrm{out}}^{*}$, using our definitions of $g(T_{\mathrm{in}})$ and $f(T_{\mathrm{out}})$

$$\frac{g\left(T_{\mathrm{in}}\right)}{f\left(T_{\mathrm{out}}\right)}=\frac{{{\hat{\mathrm{RR}}}_{\mathrm{in}}}^{\left(T_{\mathrm{in}}-T_{\mathrm{in}}^{*}\right)}}{{{\hat{\mathrm{RR}}}_{\mathrm{out}}}^{\left(T_{\mathrm{out}}-T_{\mathrm{out}}^{*}\right)}}$$

When $T_{\mathrm{out}}=T_{\mathrm{out}}^{*}$, the equation immediately above becomes

(4) $$\frac{g\left(T_{\mathrm{in}}\right)}{f\left(T_{\mathrm{out}}\right)}=\frac{{{\hat{\mathrm{RR}}}_{\mathrm{in}}}^{\left(T_{\mathrm{in}}-T_{\mathrm{in}}^{*}\right)}}{1}\;\Rightarrow k={{\hat{\mathrm{RR}}}_{\mathrm{in}}}^{\left(T_{\mathrm{in}}-T_{\mathrm{in}}^{*}\right)}$$

Given our assumption of full mediation, for an increase in outdoor temperature above heat threshold, the observed increase in mortality is driven by an increase in: (a) the number of days where regional indoor temperatures exceed the indoor heat threshold or (b) the magnitude by which regional indoor temperatures exceed the indoor heat threshold, or (c) both (a) and (b).

Therefore, to capture these possibilities, we introduce a threshold truncated indoor temperature, $T_{\mathrm{in}}^{+}$, which we calculate as

$$T_{\mathrm{in}}^{+}=\left\{\begin{array}{c} T_{\mathrm{in}},\;T_{\mathrm{in}}>T_{\mathrm{in}}^{*}\\ T_{\mathrm{in}}^{*},\;otherwise.\; \end{array}\right.$$

We define the function, h, which describes the relationship between outdoor temperature, $T_{\mathrm{out}}$, and the mean threshold truncated indoor temperature, $\bar{T_{\mathrm{in}}^{+}}$, as

(5) $$\bar{T_{\mathrm{in}}^{+}}=h\left(T_{\mathrm{out}}\right),\;T_{\mathrm{out}}\geq T_{\mathrm{out}}^{*}$$

Fifth, we assume that indoor temperatures that exceed the indoor threshold only do so by a small amount. Hence, for a small exceedance, x, the indoor heat mortality function, g, is approximately linear: ${{\hat{\mathrm{RR}}}_{\mathrm{in}}}^{x}≅1+\log\left({\hat{\mathrm{RR}}}_{\mathrm{in}}\right)\cdot x$. Thus, $\bar{g(T_{\mathrm{in}})}$ = $g(\bar{T_{\mathrm{in}}^{+}})$, such that

(6) $$f\left(T_{\mathrm{out}}\right)=\frac{\bar{g\left(T_{\mathrm{in}}\right)}}{k}=\frac{g\left(\bar{T_{\mathrm{in}}^{+}}\right)}{k},\;T_{\mathrm{out}}\geq T_{\mathrm{out}}^{*}$$

Last, we assume that a positive increase in outdoor temperature is associated with a smaller positive increase in indoor temperature: $\frac{d}{dT_{\mathrm{out}}}h\left(T_{\mathrm{out}}\right)<1$.

## 3 Proof

Under these assumptions, we now prove that, above the outdoor heat threshold, a change in indoor temperature is associated with a greater change in mortality than an equal change in outdoor temperature:

$$\frac{d}{d\bar{T_{\mathrm{in}}^{+}}}g\left(\bar{T_{\mathrm{in}}^{+}}\right)>\frac{d}{dT_{\mathrm{out}}}f\left(T_{\mathrm{out}}\right),\;T_{\mathrm{out}}\geq T_{\mathrm{out}}^{*}.$$

We start by expressing (6) as

(7) $$g\;\left(\bar{T_{\mathrm{in}}^{+}}\right)=f\left(T_{\mathrm{out}}\right)k$$

Then we substitute (5), $\bar{T_{\mathrm{in}}^{+}}=h\left(T_{\mathrm{out}}\right)$, into the immediately above

(8) $$g\left[h\left(T_{\mathrm{out}}\right)\right]=f\left(T_{\mathrm{out}}\right)k$$

We then take the derivative of (8) with respect to $T_{\mathrm{out}}$ such that

(9) $$\frac{d}{dT_{\mathrm{out}}}g\;\left[h\left(T_{\mathrm{out}}\right)\right]=\frac{d}{dT_{\mathrm{out}}}f\left(T_{\mathrm{out}}\right)k$$

and by the chain rule

(10) $$\frac{d}{dh\left(T_{\mathrm{out}}\right)}g\left[h\left(T_{\mathrm{out}}\right)\right]\frac{d}{dT_{\mathrm{out}}}h\left(T_{\mathrm{out}}\right)=\frac{d}{dT_{\mathrm{out}}}f\left(T_{\mathrm{out}}\right)k$$

Last, we substitute $h(T_{\mathrm{out}})$ with $\bar{T_{\mathrm{in}}^{+}}$ and rearrange as follows:

(11) $$\frac{d}{d\bar{T_{\mathrm{in}}^{+}}}g\left(\bar{T_{\mathrm{in}}^{+}}\right)\frac{d}{dT_{\mathrm{out}}}h\left(T_{\mathrm{out}}\right)=\frac{d}{dT_{\mathrm{out}}}f\left(T_{\mathrm{out}}\right)k$$

(12) $$\frac{d}{d\bar{T_{\mathrm{in}}^{+}}}g\left(\bar{T_{\mathrm{in}}^{+}}\right)=\frac{\frac{d}{dT_{\mathrm{out}}}f\left(T_{\mathrm{out}}\right)k}{\frac{d}{dT_{\mathrm{out}}}h\left(T_{\mathrm{out}}\right)}$$

Given that

-
(13) $$\;f\;\mathrm{is}\;monotonically\;increasing\Rightarrow \frac{d}{dT_{\mathrm{out}}}f\left(T_{\mathrm{out}}\right)>0$$
-
(14) $$\begin{array}{ccc} & & A\;\mathrm{positive}\;\mathrm{increase}\;\mathrm{in}\;\mathrm{outdoor}\;\mathrm{temperature}\;\mathrm{is}\;\mathrm{associated}\;\mathrm{with}\;a\;\mathrm{smaller}\\ & & \mathrm{positive}\;\mathrm{increase}\;\mathrm{in}\;\mathrm{indoor}\;\mathrm{temperature}\Rightarrow \frac{d}{dT_{\mathrm{out}}}h\left(T_{\mathrm{out}}\right)<1 \end{array}$$
- From (4),
K≥
(15) k≥1.
  Consider now (12)
$$\frac{d}{d\bar{T_{\mathrm{in}}^{+}}}g\left(\bar{T_{\mathrm{in}}^{+}}\right)=\frac{\frac{d}{dT_{\mathrm{out}}}f\left(T_{\mathrm{out}}\right)\cdot k}{\frac{d}{dT_{\mathrm{out}}}h\left(T_{\mathrm{out}}\right)}.$$
  Because of (13), (14), and (15),
$$\frac{d}{d\bar{T_{\mathrm{in}}^{+}}}g\left(\bar{T_{\mathrm{in}}^{+}}\right)>\frac{d}{dT_{\mathrm{out}}}f\left(T_{\mathrm{out}}\right).$$

## 4 Discussion

Using mathematical modeling, we have demonstrated that, under the assumptions set out above, a change in indoor temperature is associated with a greater change in mortality than an equal change in outdoor temperature. This supports the findings of Huang et al. (2025) and Ragettli et al. (2024), which, to our knowledge, are the only studies to have estimated an exposure–response relationship between indoor temperature and mortality, while also allowing a direct comparison with –that between outdoor temperature and mortality. Although Huang et al. (2025) suggest that this finding may be due to a longer exposure duration to indoor temperature than outdoor temperature, our work implies that it is due to the relationship between regional daily average indoor and outdoor temperature, where a positive increase in outdoor temperature is associated with a smaller positive increase in indoor temperature. These explanations are not mutually exclusive, and further debate is necessary to ensure that this finding accurately informs research and public policy.

Our results are likely to benefit studies that seek to assess the health impacts of building adaptations to protect the population against heat risks (Ibbetson et al. 2025; Taylor et al. 2021). Due to a lack of evidence for an exposure–response relationship between indoor temperature and mortality, studies of this nature have relied on the assumption that a change in indoor temperature due to an intervention will have an equivalent effect on mortality as a corresponding shift along the exposure–response relationship between *outdoor* temperature and mortality. Thus, if the intervention reduces the indoor temperature by 0.5°C, they assume that the effective outdoor temperature for that individual is also reduced by 0.5°C. On the one hand, our results suggest that this may underestimate the reduction in heat risk, given that a change in indoor temperature might be associated with a greater change in mortality than an equal change in outdoor temperature. On the other hand, this will depend on the indoor temperature pre‐intervention relative to the assumed indoor heat threshold and to the outdoor temperature. For example, if the indoor temperature pre‐intervention was below the indoor heat threshold, but the outdoor temperature was above the outdoor heat threshold, the current method would overestimate the associated health benefits.

Our findings should be interpreted with caution given our use of several key assumptions. First, we assumed that indoor temperatures fully mediate the association between outdoor temperature and mortality. Although evidence suggests those most at risk of heat death spend much of their time indoors (Almeida‐Silva et al. 2014), any time spent outdoors could lead to misattribution of heat‐related deaths to indoor temperature exposure in our model. Therefore, we recognize that spending most of the time indoors does not in itself establish full mediation. Rather, full mediation is a simplifying theoretical assumption required by the present model. Further work is required to establish how this may bias our results.

Second, we assumed that the exposure–response relationship between indoor temperature and mortality has the same J‐shaped form as the exposure–response relationship between outdoor temperature and mortality estimated by epidemiological studies (Gasparrini et al. 2022). Given that we do not know the true shape of the indoor exposure–response relationship, we cannot say how this assumption may bias our results. However, physiological evidence of the human body's response to heat would appear to support our assumption (Bouchama et al. 2024).

Third, we assumed that a positive increase in regional daily mean outdoor temperature is associated with a smaller positive increase in regional daily mean indoor temperature. We emphasize that, in line with epidemiological studies, our assumption relates to the regional average indoor–outdoor temperature relationship, rather than that of individual buildings, across which the relationship may vary substantially due to differences in physical characteristics, occupancy, and occupant behavior (Mavrogianni et al. 2012). Empirical evidence, from a variety of climates, suggests that the indoor–outdoor temperature slope is typically less than unity during the warm season (DESNZ and Li 2024; Drury 2024; Nguyen et al. 2014; Nguyen and Dockery 2016; Nicol et al. 2020; Rupp et al. 2021; Tamerius et al. 2013) and, therefore, this assumption appears to be justified. However, it is important to consider the sensitivity of our result to this assumption. For if the reverse was true, such that a positive increase in outdoor temperature was associated with a *greater* positive increase in indoor temperature, then we would find that the exposure‐repsonse relationship between indoor heat and mortality is *less steep* than that between outdoor heat and mortality.

## 5 Conclusion

In this study, we developed a novel mathematical modeling approach to demonstrate why the exposure–response relationship between indoor temperature and mortality may be steeper than that between outdoor temperature and mortality. This finding is likely to benefit researchers seeking to assess the health impact of changes in indoor temperature who, in the absence of evidence for an exposure–response relationship between indoor temperature and mortality, have typically relied on the relationship between *outdoor* temperature and mortality. Moreover, our work provides a new theoretical framework for examining  potential differences in the temperature‐mortality relationship for indoor and outdoor heat exposure in epidemiological studies. Future work could further evaluate the validity of our assumptions via analysis of both the time‐activity patterns of individuals and the indoor–outdoor temperature relationship of buildings during the warm season. Large‐scale longitudinal indoor environmental monitoring linked with health outcomes is also needed, particularly in vulnerable settings, alongside data on building characteristics, adaptation measures, and occupant behavior. This would provide an empirical basis for testing and refining the theoretical framework developed in this study.

## Disclosure

The funding sources had no role in the study design; collection, analysis, or interpretation of data; writing of the report; or the decision to submit the article for publication.

## Ethics Statement

The project was approved by the research ethics committee of University College London.

## Conflicts of Interest

The authors declare no conflicts of interest.

## Data Availability

Data sharing is not applicable to this article as no datasets were generated or analyzed during the current study.
